# Mast Cells and Their Progenitors in Allergic Asthma

**DOI:** 10.3389/fimmu.2019.00821

**Published:** 2019-05-29

**Authors:** Erika Méndez-Enríquez, Jenny Hallgren

**Affiliations:** Department of Medical Biochemistry and Microbiology, Uppsala University, Uppsala, Sweden

**Keywords:** mast cell, mast cell progenitors, allergic asthma, mast cell development, mast cell activation

## Abstract

Mast cells and their mediators have been implicated in the pathogenesis of asthma and allergy for decades. Allergic asthma is a complex chronic lung disease in which several different immune cells, genetic factors and environmental exposures influence the pathology. Mast cells are key players in the asthmatic response through secretion of a multitude of mediators with pro-inflammatory and airway-constrictive effects. Well-known mast cell mediators, such as histamine and bioactive lipids are responsible for many of the physiological effects observed in the acute phase of allergic reactions. The accumulation of mast cells at particular sites of the allergic lung is likely relevant to the asthma phenotype, severity and progression. Mast cells located in different compartments in the lung and airways have different characteristics and express different mediators. According to *in vivo* experiments in mice, lung mast cells develop from mast cell progenitors induced by inflammatory stimuli to migrate to the airways. Human mast cell progenitors have been identified in the blood circulation. A high frequency of circulating human mast cell progenitors may reflect ongoing pathological changes in the allergic lung. In allergic asthma, mast cells become activated mainly via IgE-mediated crosslinking of the high affinity receptor for IgE (FcεRI) with allergens. However, mast cells can also be activated by numerous other stimuli e.g. toll-like receptors and MAS-related G protein-coupled receptor X2. In this review, we summarize research with implications on the role and development of mast cells and their progenitors in allergic asthma and cover selected activation pathways and mast cell mediators that have been implicated in the pathogenesis. The review places an emphasis on describing mechanisms identified using *in vivo* mouse models and data obtained by analysis of clinical samples.

## The Origin of Mast Cells in Mouse and Man

The development of mast cells has predominantly been studied in mice. The first phenotypic identification of mast cell progenitors (MCps) was made in fetal mouse blood, where isolated Thy-1^lo^ c-kit^hi^ cells generated mast cells *in vitro* and could reconstitute mast cell deficient mice *in vivo* (1). *In utero*, mast cells originate from yolk sac-derived progenitors (2–4). Due to their slow turnover, yolk sac-derived mast cells to some extent remain while slowly being replaced by maturing bone-marrow derived mast cells in the adult (3, 4). In young naïve mice, mast cells in connective tissues such as in the skin and peritoneum are derived from fetal yolk sac/liver MCps (4). In other adult tissues, mast cells arise from committed MCps that under the influence of growth factors and transcriptional control differentiate into mast cells. The first identification of a mast cell precursor in adult mice was made by three independent labs (5–7). Chen et al. described a committed MCp population in the bone marrow by isolation of lineage (Lin)^−^ c-kit^+^ Sca-1^−^ Ly6c^−^ FcεRI^−^ CD27^−^ integrin β7^+^ ST2^+^ cells using fluorescence-activated cell sorting (FACS) (6). Jamur et al. used immunomagnetic isolation by two monoclonal antibodies recognizing specific surface sites on rodent mast cells to isolate CD34^+^ CD13^+^ c-kit^+^ FcεRI^−^ bone marrow cells, which developed to mast cells *in vitro* and *in vivo* (5). Meanwhile, Arinobu and colleagues demonstrated a committed MCp population in the intestine and a bipotent basophil–mast cell progenitor (BMCp) in the spleen (7). The close relationship between mast cells and basophils was supported by a study showing that isolated single granulocyte-monocyte progenitors (GMp) were capable of differentiating into both mast cells and basophils (8), which was recently confirmed by the demonstration of a BMCp population distinguished as Lin^−^ Sca-1 ^−^ c-kit^+^ integrin β7^hi^ CD16/32^hi^ cells in mouse bone marrow using single cell RNA-sequencing (9). By taking advantage of the expression of GATA-1 in eosinophils, basophils and mast cells, Drissen et al. used *Gata-1*-EGFP mice to fractionate and to identify distinct myeloid progenitors by single cell sequencing (10). This study suggested that Gata-1^+^ progenitors, defined as Lin^−^ c-kit^hi^ CD41^−^ cells with variable expression of CD16/32, have the capacity to differentiate into eosinophils, mast cells or basophils.

While the main point of hematopoiesis in adults likely occurs in the bone marrow niche, white adipose tissue (WAT) has been demonstrated to contain not only adipocytes but also a quite large fraction of immature immune/hematopoietic cells, called the stroma-vascular fraction (SVF) (11). In agreement with this, the SVF also contains MCps and mast cells (12). In a model of acute myocardial infarction, WAT-derived MCps infiltrated the heart and gave rise to an increased mast cell population at this location (13). However, the differential contribution of bone marrow-derived vs. WAT-derived MCps creating increased pools of mast cells at different sites of the body during inflammatory conditions is currently unclear.

In homeostatic conditions, committed MCps can be detected by flow cytometry in the blood (14) and peripheral tissues of naïve laboratory mice (15). The MCp population in the blood is distinguished as Lin^−^ c-kit^hi^ ST2^+^ integrin β7^hi^ CD16/32^hi^ cells (14). The MCps from the BALB/c and C57BL/6 strains differ in maturity (14). BALB/c mice have a higher proportion of FcεRI^+^ MCps in the circulation, but even the FcεRI^−^ MCp differentiated into double positive c-kit^+^ FcεRI^+^ cells *in vitro*, whereas the blood MCps in C57BL/6 mice were largely FcεRI^−^ and retained some basophil differentiation potency (14). The MCps are of lymphocyte size, contain none or a few granules and have a typical progenitor morphology. They do not stain, or stain weakly with basic dyes that typically stain mast cells metachromatically. The MCps are extremely rare, constituting only around 50 cells per 10^6^ enriched mononuclear cells in the lung and peripheral blood. However, their existence in the periphery of naïve mice was predicted years ago by limiting dilution and clonal expansion assays (16, 17).

The development of MCps into mast cells *in vivo* is largely dependent on stem cell factor (SCF), which has effects on homing, proliferation, survival and function of mast cells and their progenitors. Interestingly, local administration of SCF promotes the expansion of mast cells *in vivo* (18). The importance of SCF in mast cells is underscored by the lack of mast cells in mice lacking the expression of a functional c-kit receptor, as in Kit^*W*/*Wv*^ (19) or Kit^*W*−*sh*/*W*−*sh*^ mice (20). Nevertheless, mouse mast cells can be derived *in vitro* by culture of hematopoietic cells with IL-3 alone (21, 22).

In 2016, we identified a human MCp population defined as Lin^−^ CD34^hi^ CD117^int/hi^ (c-kit) FcεRI^+^ cells in the blood circulation (23). As with their mouse counterparts, the human MCps have an immature appearance, express mast cell specific genes and develop into mast cells *in vitro*. Interestingly, the frequency of circulating blood MCps was higher in individuals with a reduced lung function (23). For a comparison, see Table 1. When the human MCp population was investigated in patients receiving treatment inhibiting signaling through CD117 (*imatinib*) and depleting mast cells *in vivo*, the MCp population was intact (24). These results suggest that signaling through CD117 is dispensable for human MCps to develop and survive. However, for human MCps to develop into mast cells, SCF is required, as summarized in (25).

**Table 1:** Comparison of human and mouse mast cell progenitors.

**Feature**	**Mouse**	**Human**
The nucleus occupies most of the cytoplasm	+++	+++
FcεRI^+^	~100% (tissue)/ ~25–70% (blood)	100% (by definition)
Integrin β7 surface expression	+++	++
Frequency in the blood	~0.005%^*^	~0.005%^*^
Cell division in vitro^**^	+++	+

* *Of Ficoll-separated cells*.

** *In a myeloid-erythroid cytokine cocktail*.

## Mast Cell Subtypes

Rodent mast cells are classically distinguished into two different phenotypes, connective tissue-type mast cells (CTMCs) and mucosal mast cells (MMCs). The division was originally based on their respective biochemical properties, which led to different staining patterns in response to histochemical dyes and corresponded to their location in the gut (26). CTMCs have heparin glycosaminoglycan-chains attached to the serglycin proteoglycan core protein, whereas in MMCs serglycin carries chondroitin sulfate-chains (27). Apart from the location in the submucosa of the gut, CTMCs are also found in the peritoneum and skin, while MMCs predominate in the intestinal mucosa. CTMCs express relatively high levels of mouse mast cell protease (mMCP)-4,-5 (chymases) and -6,-7 (tryptases), but not mMCP-1 and-2 (chymases), whereas MMCs express mMCP-1 and -2 and not mMCP-4,-5 and -6 (28, 29). Of note, the life span of CTMCs is extensively longer than that of MMCs (30), at least in the absence of ongoing inflammation. In accordance with this, CTMCs are constitutive and at least in the young mice they are mainly derived from fetal mast cells with self-generating capabilities (4). MMCs are induced and expand upon, e.g. inflammatory stimulus.

Similarly, human mast cells are divided into two subtypes. Since the mast cells in the human lung were shown to have both heparin and chondroitin sulfate proteoglycans (31), they were classified according to their protease content. Some human mast cell populations, e.g. skin mast cells, express both tryptase and chymase (MC_TC_) corresponding to the CTMCs in rodents, whereas other mast cell populations, e.g. in the bronchial/bronchiolar epithelium, predominately lack chymase expression (MC_T_), roughly corresponding to MMCs (32, 33). Nevertheless, the classification of mast cells into these two phenotypes is really an oversimplification for mast cells in the lung. In mice, both antibody-based methods (29) and microarray data from the ImmGen project (34) suggest that lung mast cells express a much wider range of proteases than previously thought. The constitutive mast cells in the trachea and lung of mice contained mast cells immunopositive for mMCP-1 and -2, mMCP-4,-5,-6 and -7 and carboxypeptidase A3 (CPA3), whereas the induced MMCs present in the epithelium of antigen-sensitized and challenged mice expressed mMCP-1,-2 and -6,-7 (29). In accordance with this study, constitutive tracheal mast cells express transcripts for mMCP-4,-5 and -6,-7 and CPA3 (34). In humans, studies of bronchial and transbronchial biopsies from non-smokers suggest a similar situation, where MC_T_ and MC_TC_ coexist in all compartments of the lung (35). Given that, MC_T_ were more frequently found in the bronchi, bronchioles and alveolar parenchyma, whereas MC_TC_ dominated in pulmonary vessels and pleura. The MC_T_ and MC_TC_ phenotypes could also be further divided into site-specific populations, which showed specific expression patterns of, for example, FcεRI, IL-9 receptor, histidine decarboxylase (HDC) and leukotriene C4 synthase (LTC_4_-S) (35). The most intriguing finding was the presence of alveolar mast cells that lack surface expression of FcεRI.

## Mast Cell Accumulation in the Lung and the Mechanisms Behind

### Mast Cell Accumulation in the Lung of Asthma Patients

**Figure 1:**
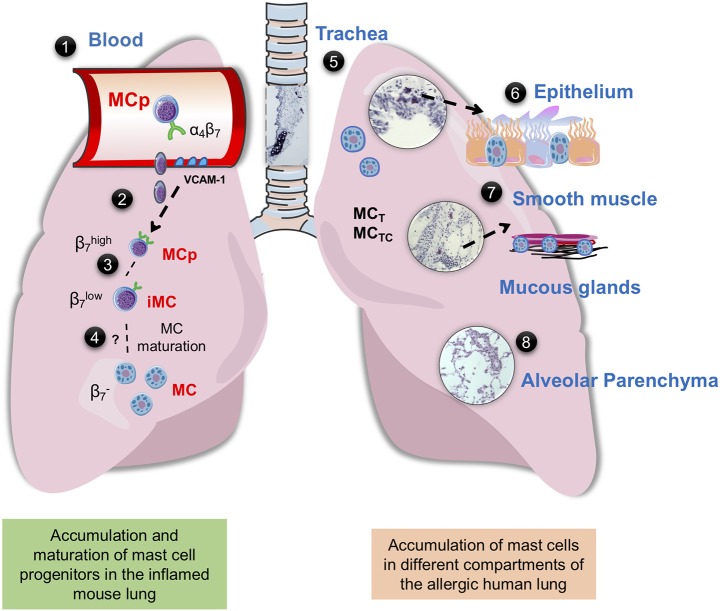
Mast cells in mouse and human lung. **(1)** Integrin-β7^+^ mast cell progenitors (MCps) are found in mouse and human peripheral blood (14, 23). **(2)** In mice with acute allergic airway inflammation, MCps are recruited to the lungs in a process dependent on α4β1 and α4β7 integrins on the MCp and on VCAM-1 expressed in the endothelium (36). **(3)** After the acute phase, three mast cell (MC) populations can be identified by flow cytometry, MCps expressing high levels of integrin β7, immature/induced MCs (iMCs) expressing intermediate levels of integrin β7, and mature MCs (37, 38). **(4)** The iMC gradually loses the expression of integrin β7 and mature, thereby expanding the resident lung MC population. **(5)** In the mouse trachea and in the proximal bronchi of unprovoked mouse airways, MCs express the MC proteases mMCP-1 and-2, mMCP-4,-5-6, and-7 and CPA3, while the MCs induced by allergic airway inflammation located in the bronchovascular bundles of the lung and the epithelial lining of the large bronchi express mMCP-1,-2 and -6,-7 (29). In the human lung MC_T_ and MC_TC_ coexist, MC_T_ is more frequently found in the bronchi, bronchioles and alveolar parenchyma, whereas MC_TC_ dominates in pulmonary vessels and pleura (35) **(6)** In the human bronchi, patients with “Th2-high” asthma have an increased number of intraepithelial MCs (39). Genetic analyses suggest that the MCs in this location mainly express tryptase and CPA3 (39–41). **(7)** The number of MCs are increased in the airway smooth muscle of asthma patients (42, 43). In diseased asthma patients, there are an increased number of mast cells in the distal airway, especially in the smooth muscle and mucous glands (44). **(8)** Uncontrolled atopic asthmatics have a high number of mast cells in the alveolar parenchyma (45). The histology pictures shown are from hematoxylin/eosin-stained lung sections of house dust mite-sensitized wild-type BALB/c mice obtained from our unpublished experiments.

Several studies suggest that the presence or accumulation of mast cells at certain compartments of the lung are pathological features of allergic asthma (Figure 1). An increased number of mast cells were found in the airway smooth muscle of asthma patients in comparison to controls or subjects with eosinophilic bronchitis (42). The number of mast cells was also higher in the smooth muscle of allergic asthmatics in relation to non-allergic asthmatics (43). In support of these studies, isolated human bronchi with the ability to contract in response to allergens had a higher number of smooth muscle-associated mast cells than unresponsive human bronchi isolates (46). Moreover, an increased number of mast cells was found in the distal airways of subjects with non-fatal and fatal asthma compared to non-asthmatic controls (44). In the distal airways, the greatest increase in mast cells was found within the smooth muscle and mucous glands. In another study, the same authors found an increased percentage of degranulated mast cells in the mucous glands from fatal asthma in comparison to non-fatal asthma and controls, suggesting that mast cells are highly activated in fatal asthma (47). In biopsies from patients with severe asthma, the number of MC_TC_ and the MC_TC_/MC_T_ ratio in the small airways were higher compared to normal subjects (48). However, a positive correlation between MC_TC_ in the region of small airways/alveolar attachments and lung function was found (48), suggesting a protective role of this subtype of mast cells.

Increased mast cell numbers are also found in other lung compartments of asthma patients. For example, sputum samples from asthmatics more often showed expression signatures of mast cell-specific protease genes (*TPSAB1* and *CPA3*) and higher expression of these genes than samples from healthy subjects (49). In accordance with this, genetic analyses of samples from epithelial brushings have revealed increased gene expression of mast cell tryptase (*TPSAB1, TPSB2*) and *CPA3* (but not *CMA1*, coding for chymase) in asthma patients (39–41). In the study by Singhania et al., a twofold increase in the number of intraepithelial mast cells was found in patients with “Th2-high” asthma compared to those characterized as “Th2-low” (39). Moreover, the presence of intraepithelial mast cells in the Th2-high asthma patients predicted responsiveness to inhaled corticosteroids. In a study of severe asthma, the proportion of MC_TC_ among all mast cells was higher in patients with severe asthma compared to those with mild asthma (50). A higher proportion of MC_TC_ was also found in the bronchi of uncontrolled atopic asthmatic subjects, and overall a higher number of mast cells was found in the alveolar parenchyma, paralleled by an increase in their expression of FcεRI (51). In a follow-up study, patients with mild atopic asthma were found to have an elevated number of alveolar mast cells with increased expression of FcεRI in comparison to healthy controls and non-asthmatic allergic rhinitis patients (45). Although the above-mentioned studies were performed on adults, another study demonstrated that submucosal mast cells are more frequent in bronchial biopsies from symptomatic children with severe asthma than in those with few symptoms (52). To summarize, mast cells accumulate in the smooth muscle, bronchial epithelium and alveolar parenchyma of patients with allergic and severe asthma, thereby presumably increasing the detrimental consequences of mast cell activation in the allergic lung. However, in some compartments of the lung, there might be an advantage to having chymase-expressing mast cells.

### Identified Mechanisms and Factors Involved in Mast Cell Accumulation in the Lung

To address whether the increase in lung mast cells in patients with allergic asthma is due to the recruitment of MCps to the lung or a result of local proliferation of resident mast cells, mouse models have been used. In early studies, lung MCps were quantified by a limiting dilution and clonal expansion assay in an ovalbumin (OVA)-model of allergic airway inflammation (36). In OVA-sensitized and challenged mice, the number of lung MCps was around 30 times higher 1 day post-challenge than in control mice. The OVA-induced increase in lung MCps was absent in mice genetically deficient of endothelial VCAM-1 and in wild-type mice treated with blocking antibodies targeting VCAM-1, α4-, β1-, or β7-integrins during the challenge phase (36). This suggests that allergic airway inflammation induces the recruitment of MCps to the lung. After the acute phase, the accumulation of lung MCps gradually led to the appearance of toluidine blue^+^ mast cells in the epithelium of the trachea and the lung parenchyma (53, 54). Moreover, the OVA-induced recruitment of MCps to the lung was dependent on the presence of CD11c^+^ cells (54) and CD4^+^ cells (55), demonstrating that an adaptive immune response is required to stimulate this process.

In an effort to study a possible chemokine component in the antigen-induced recruitment of MCps to the lung, mice lacking CCR3 or CCR5 were used to test whether any of these chemokine receptors were involved in this process. However, CCR3^−/−^ and CCR5^−/−^ mice had an intact OVA-induced recruitment of MCps to the lung (53). Nevertheless, the OVA-induced recruitment of MCps to the lung was partly dependent on the presence of CXCR2 in stromal cells in the lung. Mice lacking CXCR2 had a reduced inflammation-induced upregulation of VCAM-1 on the lung endothelium, which could explain the reduced recruitment of MCp to the lung (53). CCR2-deficient mice also had a reduction in OVA-induced recruitment of MCps to the lung (56). Nonetheless, this was likely due to unidentified stromal defects and not to MCps lacking CCR2 *per se* (56). Therefore, any chemokine component required for the recruitment of MCps to the lung remains unknown.

The role of cytokines in OVA-induced recruitment of MCps to the lung has also been a matter of investigation. Interestingly, the OVA-induced recruitment of MCps to the lung occurs independently of genetic ablation of IL-4, IL-4Rα chain, STAT-6, IFN-γ, and IL-12 and antibody-mediated neutralization/blocking of IFN-γ, IL-3, IL-4, IL-5, IL-6, IL-13, IL-17A, IL-12p40, or IL-12p40Rβ1 during the challenge phase (55). However, IL-9 deficiency or IL-9 antibody neutralization efficiently prevented the OVA-induced recruitment of MCps to the lung. In an effort to identify the source of IL-9, we also found that genetic ablation of CD1d or blocking CD1d during the challenge phase inhibited the OVA-induced recruitment of MCps to the lung, but genetic ablation of invariant NKT cells (Jα18 deficient mice) had an intact infiltration of MCps to the lung (55). As blocking CD1d in IL-9-deficient mice or neutralizing CD1d in IL-9-deficient mice did not further inhibit the OVA-induced recruitment of MCp to the lung, type 2 NKT cells may provide or elicit IL-9 production (55). The importance of IL-9 in the accumulation of lung mast cells during allergic airway inflammation was also highlighted in a study where adoptive transfer of Th9 cells followed by challenge with OVA and TSLP increased the mast cell numbers estimated by histological analyses (57). Treatment with an anti-IL-9 antibody blocked the mast cell accumulation in both the adoptive transfer model and in an OVA sensitization and challenge model (57). In the same paper, decreased mast cell numbers were found in mice with PU.1-deficient T cells, which have reduced IL-9 levels in house dust mite (HDM)-induced allergic airway inflammation.

The importance of IgE for the survival of lung mast cells was demonstrated in a model of *Aspergillus Fumigatus*-induced allergic airway inflammation (58). However, no defect in the *Aspergillus*-induced recruitment of MCps to the lung could be detected in mice lacking IgE. Nevertheless, we found that the number of lung MCps increased significantly when sensitized wild-type mice were challenged with IgE-antigen immune complexes compared to control mice given the same dose antigen alone (59). The stimulating effect of IgE-immune complexes on the recruitment of MCps to the lung was lost in FcRγ-deficient mice, but not in CD23-deficient mice, indicating that MCp recruitment can be potentiated by Fc receptor-mediated activation (59). Thus, IgE-immune complex formation and IgE alone impacts the recruitment of MCps to the lung and the survival of lung mast cells, respectively.

The technical advances in multi-color flow cytometry have made it possible to distinguish different mast cell populations simultaneously. Using a prolonged protocol of OVA-induced allergic airway inflammation, three lung mast cell populations could be identified, MCps expressing high levels of integrin β7, resident mature mast cells and emerging/induced mast cells (37). In parallel, we demonstrated that influenza infection in mice induced the recruitment of MCps to the lung, which later gave rise to mast cells with intermediate expression of integrin β7 (immature/induced) and even later increased the number of mature mast cells with low expression of integrin β7 (38). While the recruitment of MCps induced by allergic lung inflammation was dependent on an adaptive immune response (54, 55), the influenza-induced MCp recruitment to the lung was due to the induction of innate immune responses (60). As respiratory infections commonly cause exacerbations of asthma symptoms, we think it is intriguing that influenza infections induces mast cell accumulation in the mouse lung.

### Lung Physiology in Mouse and Humans

Mice have frequently been used to model allergic asthma with the aim of investigating the mechanism behind the disease. When interpreting the results from mouse models of allergic airway inflammation it is necessary to understand that the lungs of mice and humans have anatomical and physiological similarities and differences. The right lung of humans and mice consists of five lobes. The left lung in humans consists of two lobes, whereas mice only have a single lobe on the left side (61). Another difference is that mice have monopodial branching whereas humans have dichotomous branching of the airways. Further, the intrapulmonary bronchi in mice lack cartilage, which suggests that the difference between bronchi and bronchioles is less obvious in mice than in humans. The number of goblet cells and submucosal glands is lower in mice, at least in laboratory mice that have not been infected by pathogens or subjected to a disease model. Moreover, whereas smooth muscle cell bundles populate the connective tissue surrounding the respiratory bronchioles in humans, mice have very few respiratory bronchioles that lack a smooth muscle layer.

When comparing human and naïve mouse lungs there is also an apparent difference in the quantity of mast cells and their distribution. In human lungs, mast cells are located throughout the airways and in the parenchyma, while in naïve mice mast cells are mainly found in the trachea and in the central airways (62). However, in mouse models of allergic asthma and influenza infection, the induced lung mast cells accumulate at places where they are not usually found such as in the epithelium, surrounding bronchioles, in the perivascular space and in the alveolar parenchyma (38, 53, 54, 63). Although allergic airway inflammation in mice induced by e.g. OVA and HDM stimulates airway hyperresponsiveness (AHR) to methacholine *in vivo*, these models rarely induce antigen-induced bronchoconstriction that can be measured *in vivo*. Importantly, neither lung mast cell expansion nor lung function have been analyzed in the majority of published studies of allergic airway inflammation in mice. Nevertheless, a high dose of HDM (125μg) given intranasally on five consecutive days/week over 3 weeks to induce allergic airway inflammation also induced mast cell expansion and an increase in the mast cell specific mediator mMCP-1, along with HDM-induced bronchoconstriction (63). Importantly, the HDM-induced bronchoconstriction was abrogated in Kit^*W*−*sh*/*W*−*sh*^ mice.

In contrast, when isolated mouse trachea from mice with allergic airway inflammation is analyzed *ex vivo*, antigen-induced contractions can be measured using OVA as the antigen (64). The antigen-induced contractions *ex vivo* are also abrogated in Kit^*W*−*sh*/*W*−*sh*^ mice (64). A possible reason for the discrepancy between the lack of OVA-induced bronchoconstriction *in vivo* and the presence of OVA-induced contraction in isolated airways may be that the majority of mast cells are found around the central airways and hence it is easier to measure their responsiveness to antigen in isolation (*ex vivo*). Nevertheless, it is currently unclear whether the reason behind why it is so hard to observe antigen-induced bronchoconstriction *in vivo* is only due to a less expanded lung mast cell population with lower doses of antigen and more acute protocols, or whether there are other more profound differences in lung physiology between the species that also play a role.

More often *in vivo* studies of allergic airway inflammation in mice measure AHR with increasing doses of methacholine. There are examples of protocols that bypass the dependence of mast cells for AHR, e.g. (65–67), while other protocols find that mast cells, or a mast cell mediator, are necessary for a full-blown AHR, e.g. (68–71). Moreover, when Fuch et al. compared Kit^*W*−*sh*/*W*−*sh*^ mice with Kit^*W*−*sh*/*W*−*sh*^ mice reconstituted by bone marrow-derived mast cells (BMMCs) to wild type sensitized and challenged with OVA, the reconstituted mice had a higher density of mast cells which were distributed differently compared to the wild-type mice (72). This resulted in an increased AHR in the Kit^*W*−*sh*/*W*−*sh*^ mice reconstituted with BMMCs as compared to wild-type mice treated in parallel. We speculate that this conundrum reflects the situation in human asthma, i.e. that not all phenotypes of asthma have a significant mast cell component, while some do.

## The Role of Mast Cells in Allergic Airway Inflammation

Numerous studies have shown how allergen challenge induces mast cell activation and changes in the lung function in sensitized mice. However, most of the studies have used OVA as an allergen. Presumably, HDM or other human allergens are more relevant to use for studies of allergic airway inflammation in mice since they are complex allergens that activate innate receptors and induce secretion of alarmins in addition to inducing adaptive immune responses.

FcγR-mediated activation of mast cells was demonstrated to be required for the development of AHR and inflammation in an OVA model of allergic airway inflammation using mast cell-deficient Kit^*W*/*Wv*^ or Kit^*W*−*sh*/*W*−*sh*^ mice (68, 69). Later, mast cell-derived TNF-α was implicated in mediating these features of allergic airway inflammation and AHR (70, 73). The Kit^*W*−*sh*/*W*−*sh*^ strain was also studied in a model of HDM-induced allergic airway inflammation (74). In this study, the Kit^*W*−*sh*/*W*−*sh*^ mice developed allergic airway inflammation but had reduced plasma IgE levels and bronchoalveolar lavage (BAL) eosinophils. However, lung function was not analyzed in this study (74). HDM has also been demonstrated to induce an increase in the levels of mMCP-1 in serum 30 min after a single intratracheal challenge (75). Prophylactic treatment with the mast cell stabilizer cromoglycate 1 h before HDM challenge suppressed the induced levels of mMCP-1, and when HDM was given repeatedly to induce allergic airway inflammation, cromoglycate-treatment before each HDM administration reduced the inflammatory response.

Possibly also indicating the involvement of IgE-antigen-mediated activation of mast cells in AHR, FcεRI-deficient mice had reduced AHR to methacholine in OVA-induced allergic airway inflammation (76). In a subsequent study, FcεRI-deficient mice exposed to nebulized OVA demonstrated diminished tracheal responses to electric field stimulation, which was normalized in FcεRI-deficient mice adoptively transferred with wild-type BMMCs but not after adoptive transfer of IL-13-deficient BMMCs (77). This suggests a role for IgE-antigen-mediated mast cell-derived IL-13 in antigen-induced bronchoconstriction. On the other hand, allergic airway inflammation and AHR were unperturbed in IgE-deficient mice in a model induced by repeated intranasal exposure to *Aspergillus fumigatus* extract (65). Still, pre-treatment with crosslinking monoclonal anti-mouse IgE enhanced the bronchoconstriction induced by methacholine in wild-type but not in mast cell-deficient (Kit^*W*/*Wv*^) naive mice (78). However, as c-kit mediates early development of cell linages other than mast cells, the phenotype observed in *Kit*-dependent mouse models of mast cell deficiency cannot be securely associated only to mast cells (79, 80). The results obtained from *Kit*-dependent mast cell-deficient mouse models need to be re-evaluated using the new transgenic mouse strains that do not depend on a functional c-kit for their mast cell deficiency. There is a risk that the scientific community has overestimated the role of mast cells by trusting the data that have been generated using the *Kit*-dependent mouse strains. In addition, reconstitution experiments performed with BMMCs need to be interpreted with care as BMMCs do not fully replicate the natural lung mast cell phenotype. In re-constitution experiments of mast cell-deficient mice, BMMCs may end up at a higher (or lower) density and be routed to places other than where they normally exist in wild-type mice (72). A more thorough discussion about *Kit*-dependent mast cell-deficient models, their advantages and disadvantages and discussion about *Kit*-independent models can be found in (79, 80).

Few studies have used *Kit*-independent mouse models to study the role of mast cells in experimental asthma. The Mas-TRECK and Bas-TRECK mice carry a diphtheria toxin (DT)-based conditional deletion system using intron regions of the *Il4* gene, which constitute enhancer elements that drive IL-4 production in mast cells and basophils (81). DT treatment in the Mas-TRECK mice results in loss of mast cells and basophils, whereas DT treatment in the Bas-TRECK mice leads to basophil-specific depletion. In a model of OVA-induced allergic airway inflammation, DT treatment before the challenge phase reduced AHR, which was accompanied by a remarkable reduction in histamine levels in Mas-TRECK but not in Bas-TRECK mice (81). Therefore, the study suggests that mast cells are critical for full-blown AHR while basophils are dispensable. There are also other recently constructed mouse strains which more specifically deplete mast cells but also reduce basophil numbers. One example is, the Cpa3^*Cre*/+^ strain, which takes advantage of the ability of Cre recombinase to induce toxicity if the expression is high as in CPA3-expressing mast cells (82). The Cpa3^*Cre*/+^ mice lack mast cells but have a normal number of other cell linages except for a reduction in the number of basophils. The Cpa3^*Cre*/+^ mice are currently used to re-evaluate the role of mast cells in allergic airway inflammation.

Nevertheless, c-kit (CD117) is also vital for human mast cell development and survival. Therefore, a recent proof-of-principal asthma study tested the effects of *imatinib*, a tyrosine kinase inhibitor designed to target ABL (Abelson murine leukemia viral oncogene homolog 1) for treatment of chronic myelogenous leukemia (CML), which also inhibits several other tyrosine kinases including c-kit (83). In this randomized, double-blind, placebo-controlled study, severe asthmatics were treated with *imatinib* for 24-weeks. The *imatinib* treatment reduced AHR and decreased the number of mast cells in endobronchial biopsies and tryptase levels in serum, suggesting that mast cells contribute to the pathogenesis of severe asthma and highlighting the importance of c-kit in human mast cells.

## IgE-antigen-Mediated Activation

Mast cells and basophils express FcεRI as a complex consisting of an α-chain, a β-chain and two γ-chains. In humans, FcεRI is also present as a complex composed of the α chain and two γ-chains in Langerhans cells (84), dendritic cells (85), platelets and megakaryocytes (86), neutrophils (87), monocytes (88) eosinophils (89) and airway smooth muscle cells (90).

The trimeric receptor αγ2 on human blood dendritic cells and monocytes may function as a regulator of serum IgE levels by receptor internalization (91). In mice, the tetrameric form of FcεRI has also been demonstrated in mouse nerve cells (92, 93). However, the receptors were thought not to be expressed on the cell surface in the absence of the β chain (94, 95). Nevertheless, expression of the trimeric form of FcεRI has been found in dendritic cells after virus infection (96).

Activation of mast cells by IgE-antigen activates several intracellular signaling pathways that lead to the secretion of mediators which occur in waves and mediate potent biological effects (97, 98). The mast cell-mediators activate endothelial, epithelial, and smooth muscle cells, neurons and other immune cells, thereby inducing the influx of inflammatory cells and changes in lung function (99). The first wave of mast cell mediators released after IgE-antigen-mediated activation are preformed granule-associated mediators, which are released within 5 min after the antigen contact. The composition of granule-associated mediators varies between species and differs between mast cell subtypes and localization. However, they are generally composed of histamine and (or) serotonin, proteoglycans and proteases. In the second wave of mediators after IgE-antigen-mediated mast cell activation, newly formed lipid mediators generated from arachidonic acid, are released (100). Next, cytokines and chemokines, which require gene transcription and synthesis, are secreted hours after antigen contact. For a detailed description of the molecular events which follow IgE-antigen-mediated mast cell activation, see (101).

The presence of specific IgE in serum is a key feature of allergic asthma. Also, high total IgE levels in serum are strongly related to increased risk of asthma and are correlated with AHR (102, 103). Moreover, the success of *omalizumab*, a humanized monoclonal anti-IgE antibody, which leads to improvements in symptoms and quality of life, and reduces virus-induced exacerbations (104), stresses the significance of IgE in the pathogenesis of at least some subtypes of asthma (105). Importantly, *omalizumab* reduces mast cell degranulation and FcεRI-receptor expression on mast cells and basophils in patients with moderate to severe allergic asthma (106). IgE alone is also relevant for mast cells in the asthmatic lung. For example, monomeric IgE can induce the secretion of cytokines in BMMCs, which by an autocrine mechanism enhance their survival (107). However, individual IgE molecules vary in their ability to induce cytokine production and survival (108). Several studies demonstrating the effects of IgE alone on mast cells are summarized in (109). Interestingly, the cytokinergic activity of monomeric IgE is enhanced in the presence of cytokines such as IL-4 (110). Therefore, the capability of monomeric IgE to promote mast cell survival may be significant in the asthmatic lung.

## IgE-independent Activation of Mast Cells in Asthma

**Figure 2:**
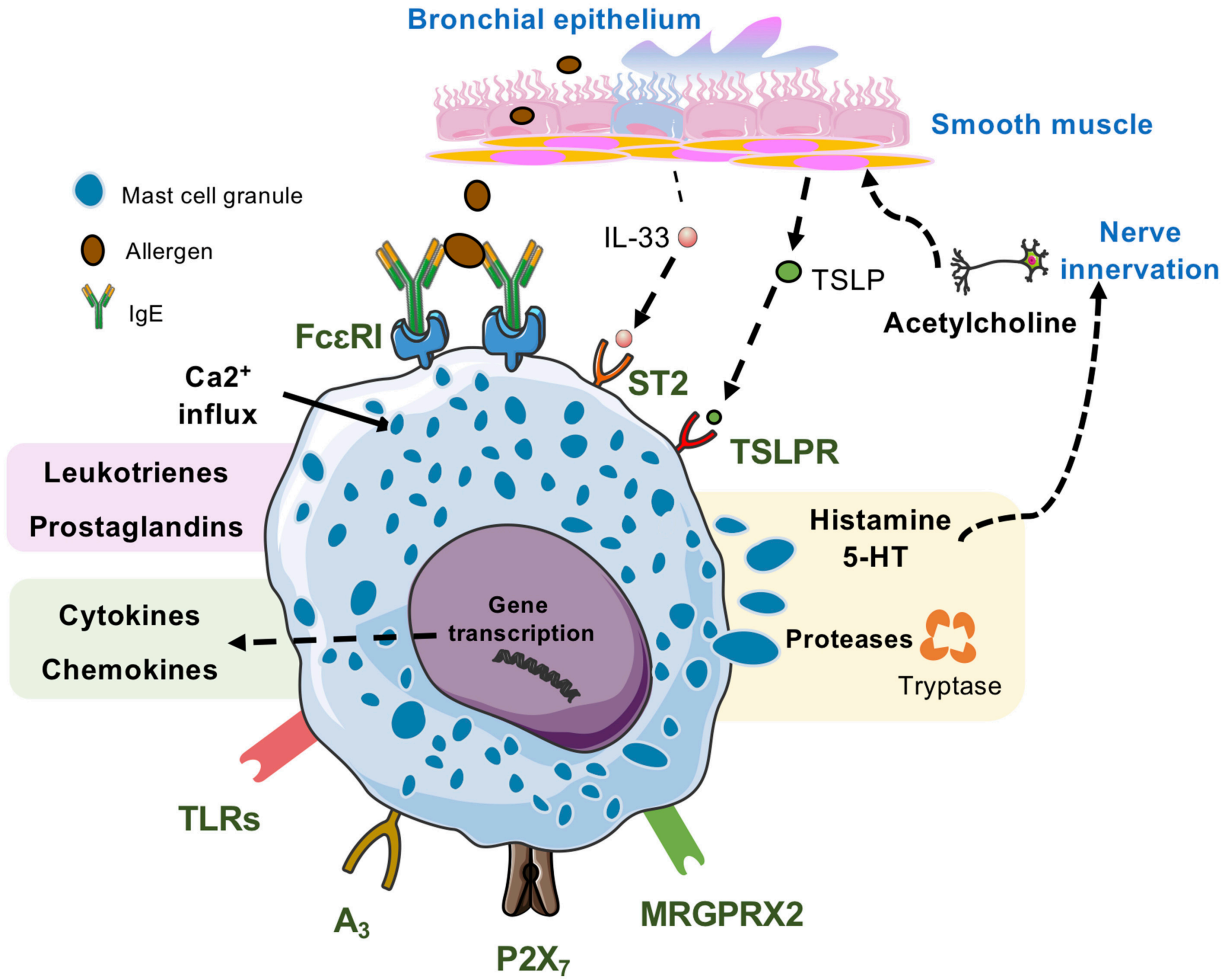
Lung mast cells can be activated by many kinds of stimuli. IgE/antigen-mediated activation of FcεRI triggers exocytosis of granular compounds, a rapid generation and release of lipid mediators such as leukotrienes and prostaglandins and the synthesis and release of cytokines and chemokines, which occurs hours after the activation event. Mast cell activation via MRGPRX2, the adenosine A3 receptor, and the ATP receptor P2X_7_ also triggers release of all three types of mast cell-derived compounds. Activation via TLRs generally triggers synthesis and release of cytokines and chemokines, and some TLRs may also trigger release of lipid mediators. However, this mode of mast cell activation does not induce degranulation. IL-33, which activates the ST2 receptor complex potentiates IgE/antigen-mediated degranulation in human mast cells but not in mouse mast cells. Alone, IL-33 triggers synthesis and release of cytokines and chemokines. In mice, TSLP acting via TSLPR promotes mast cell development, whereas in human mast cells TSLP potentiates IL-33-stimulated secretion of type 2 cytokines and chemokines.

Mast cells can be activated by several IgE-independent pathways. The possible activation mechanisms differ between mast cell subpopulations, their location in the body and the microenvironment. The IgE-independent stimuli include pathogen-associated molecular patterns (PAMPs) such as endotoxin, and products derived by the innate immune system such as complement components, cytokines and endogenous peptides (98). Here, we will summarize selected IgE-independent mast cell activation pathways which may be related to allergic asthma (Figure 2).

### MAS-Related G Protein-Coupled Receptor X2 (MRGPRX2)

Pseudo-allergic reactions typically occur in response to basic substances such as compound 48/80 or cationic peptide drugs. These reactions share characteristics with allergic responses induced by IgE-mediated responses, but trigger-specific IgE molecules are not detected (111). In 2015, MRGPRX2 (MAS-related G protein-coupled receptor X2) and the mouse ortholog MRGPRB2 were described as the main receptors involved in pseudo-allergic reactions due to their interaction with cationic drugs, which led to mast cell activation (112). In mice, *MrgprB2* transcripts were only found in CTMCs (112). The transcript levels of *MRGPRX2* are higher in human skin mast cells than in lung mast cells (113, 114). Nevertheless, the expression of MRGPRX2 on mast cells and the number of MRGPRX2^+^ mast cells are higher in lung biopsies from patients who died from asthma-related causes than in lung biopsies from patients who died from other causes (115).

Substance P (SP) was one of the identified ligands of MRGPRX2 (112). SP is elevated in BAL and sputum in asthma patients compared to healthy controls (116, 117) and is further increased in BAL immediately after allergen provocation (116). Therefore, one possibility is that MRGPRX2 is involved in a positive feed-back loop where mast cells are activated via e.g. allergen release histamine, which activates neurons to produce cationic peptides such as SP, which amplifies mast cell activation. The activation of MRGPRX2 may also be related with exacerbations of asthma symptoms that occur in connection with viral respiratory infections (118). Several respiratory viruses trigger the secretion of anti-microbial peptides such as β-defensins from epithelial cells (119, 120). β-defensins activate mast cells trough MRGPRX2 (121) and may thus contribute to virus-induced asthma exacerbations. To conclude, MRGPRX2-mediated activation of mast cells may contribute to the pathogenesis of asthma.

### Toll-Like Receptors (TLRs)

Mast cells are specialized in sensing external pathogens by recognition of pathogen-associated molecular patterns (PAMPs) and are equipped to respond to tissue damage by recognition of danger-associated molecular patterns (DAMPs) or alarmins (122). Interestingly, several allergens have the ability to stimulate toll-like receptors (TLRs), and these receptors may thus play a role in asthma development. For example, HDM extracts contain e.g. lipopolysaccharide (LPS) and proteins like Der p2 (*Dermatophagoides pteronyssinus*) mimicking MD-2, resulting in TLR4 activation (123). Mouse mast cells express several TLRs, and the expression pattern seems to differ between the mast cell subtypes, e.g. *Tlr*1-9 mRNA was detected in both MMC-like and CTMC-like BMMC but the level of *Tlr*3-5 expression differed (124). Human peripheral blood-derived mast cells express mRNA and protein for TLR1-4 and TLR6-8, and responded to double-stranded RNA stimulation via TLR3 by producing type I interferons (IFNs) (109). The expression of TLR2 was confirmed in isolated human lung mast cells by western blot, and *in vitro* stimulation with lipoteichoic acid (a TLR2 agonist) led to down regulation of FcεRI expression and decreased IgE-antigen-mediated mast cell degranulation (125). The expression of TLR4 has also been confirmed in human lung mast cells (126). In the same study, LPS stimulated release of TNF-α in peripheral blood-derived mast cells, and after pre-incubation with IFN-γ, LPS induced the expression of anti-viral genes (126). *In vivo*, TLR4-mediated mast cell activation was demonstrated to enhance eosinophilia and cytokine release in an OVA-model of allergic airway inflammation using mast cell-deficient (Kit^*W*/*Wv*^) mice and reconstitution experiments (127). Furthermore, intranasal administrations of poly I:C expand the number of lung MCps in a TLR3-dependent fashion (60). To summarize, the activation of pattern recognition receptors may directly or indirectly induce or modulate mast cell responses in the allergic lung.

### The Alarmins IL-33 and TSLP

Inhaled allergens and respiratory viruses induce the release of alarmins such as IL-33 and thymic stromal lymphopoietin (TSLP) from airway epithelial cells (128). IL-33 is a member of the IL-1 family of cytokines, which primarily binds to the IL-1 receptor family member ST2. However, the IL-33/ST2 complex assembles with the IL-1 receptor accessory protein (IL-1RAcP), which is needed for signal transduction (129). The loci for *IL1RL1* (ST2 gene) and IL-33 contains a single nucleotide polymorphism that was associated with asthma in a large-scale genome-wide association study (130). Moreover, IL-33 expression was higher in lung samples from patients with severe asthma than in those with mild asthma and IL-33 expression was mainly localized to epithelial and endothelial cells, neutrophils, fibroblast and mast cells (131). Another source of IL-33 in the airways of asthma patients are the smooth muscle cells (132). Interestingly, there was a strong inverse correlation between the concentration of IL-33 in BAL and lung function (pre-bronchodilator FEV_1_) (133).

Human *in vitro*-derived mast cells from cord and peripheral blood express ST2 (134). *In vitro*, IL-33 accelerates mast cell maturation of CD34^+^ cells and induces the secretion of Th2 cytokines and chemokines (134). Moreover, pre-treatment with IL-33 increases the number and magnitude of degranulating *in vitro*-derived mast cells in response to a crosslinking anti-IgE antibody (135). IL-33 increases the survival of human skin mast cells by upregulation of the antiapoptotic protein, B-cell lymphoma-X large (BCLXL) (136). In cord blood-derived mast cells, IL-33 alone promoted adhesion to fibronectin and production of IL-8 and IL-13 (137). In mice, lung mast cells and their progenitors express ST2 protein on the cell surface and intranasal administrations of IL-33 induce an ST2-dependent increase in lung MCp (59, 60).

The role of IL-33 in the context of allergic airway inflammation has been studied *in vivo*. Generally, mice lacking ST2 and wild type mice administrated with ST2 blocking antibodies have reduced allergic airway inflammation (138). IL-33 induces an asthma-like phenotype in *Rag2* ^−/−^ mice, which lack mature lymphocytes, demonstrating that adaptive immune responses are not required to induce an asthma-like phenotype (139). Further, in a new humanized mouse model (NOG IL-3/GM-CSF), administration of human IL-33 induced an asthma-like phenotype mediated by human IL-13 (140). The main producers of IL-13 in this setting were T cells and mast cells. Further, IL-33 given before the allergen challenge potentiated AHR in wild-type but not in mast cell-deficient Kit^*W*−*sh*/*W*−*sh*^ mice or mice treated with *ketanserin* (a nonselective 5-HT_2_R antagonist with high affinity also for H1R) in a OVA model of allergic airway inflammation (141). In contrast, in papain-induced airway inflammation, which promotes IL-33 production and increased mast cell numbers in the lung, mast cell-deficient (Kit^*W*−*sh*/*W*−*sh*^) mice had an exacerbated type 2 inflammatory response (142). The suppressive effect of mast cells in this experimental set-up was explained by the observation that IL-33-activated mast cells produced the IL-2 necessary for the expansion of CD4^+^CD25^+^Foxp3^+^ regulatory T cells that were inhibiting the type 2 inflammation by production of IL-10. Altogether, there is substantial evidence that the IL-33-ST2 pathway is involved in allergic asthma and that this pathway interacts with mast cells.

TSLP is another alarmin released from epithelial cells as a DAMP signal after allergen exposure. This IL-7-like cytokine binds to the TSLP receptor (TSLPR), which shares the alpha chain with the IL-7 receptor (143, 144). The expression of TSLP was first associated with acute and chronic dermatitis (145). In human bronchial biopsies from asthmatics, *TSLP* transcripts were increased in epithelial cells and in the submucosa (146). Interestingly, the number of epithelial cells or submucosal cells expressing *TSLP* was inversely correlated with FEV_1_ (% of predicted) (146). TSLP was also increased in serum from asthmatics compared to healthy controls or patients with chronic obstructive lung disease (COPD) (147). In mice, the expression of TSLP is increased in allergic airway inflammation and TSLPR-deficient mice have an attenuated type 2 response (148). Moreover, overexpression of TSLP in epithelial cells in the mouse lung produced a spontaneous asthma-like phenotype (148). TSLP^−/−^ mice have reduced numbers of mast cells in the intestine, kidney, nasal mucosa, skin, liver and lung, suggesting that TSLP is involved in mast cell development (149). In support of that, IL-3-induced proliferation and differentiation of BMMCs is blocked by neutralization of TSLP (149).

TSLP expression has also been demonstrated in human bronchial and submucosal mast cells as well as in the epithelium and airway smooth muscle (146, 150). Airway smooth muscle cells and human mast cells in this location expressed TSLPR (150). Further, the percentage of TSLP^+^ mast cells in bronchial samples was increased in asthmatic airways compared to healthy controls (151). In the same study, activation of human peripheral blood-derived mast cells by a crosslinking anti-IgE antibody induced TSLP expression, which was further enhanced by pre-incubation with IL-4. Moreover, TSLP potentiates the IL-33-stimulated secretion of type 2 cytokines and chemokines in human mast cells derived from peripheral blood or cord blood (134). The importance of TSLP in asthma is highlighted by clinical studies using a monoclonal anti-TSLP antibody. In 2014, an anti-TSLP antibody showed efficacy in several measures of allergen-induced early and late asthmatic responses including improved FEV_1_ (% of predicted) (152). Furthermore, anti-TSLP treatment has been shown to reduce the frequency of asthma exacerbations and improved FEV_1_ (% of predicted) in a randomized, double-blind, placebo-controlled trial of uncontrolled asthmatics with moderate to severe asthma (153). Altogether, the present data suggest that TSLP influences the development of mast cells and that mast cell-derived TSLP may contribute to allergic asthma.

### Purinergic Signals (ATP, Adenosine)

Adenosine tri-phosphate (ATP) is a danger signal to the immune system when released to the extracellular milieu by many different cells types. Extracellular ATP is sensed by the class 2 purinergic P2Y and P2X receptors (154). In the extracellular space, ATP is quickly hydrolyzed by nucleoside triphosphate diphosphohydrolase (NTPDase or CD39) to adenosine mono-phosphate (AMP) via adenosine di-phosphate (155). Allergen challenge triggers an increase in ATP levels in BAL from asthma patients and mice with experimentally induced allergic airway inflammation (156). In the same study, neutralizing ATP by the ATP-hydrolyzing enzyme apyrase or inhibition with a broad-range inhibitor of P2 receptors before OVA challenge blocked type 2 inflammation and AHR.

That rodent mast cells are activated and degranulate in response to ATP has been known for several decades (157, 158). In an acute mouse model of colitis, ATP-mediated mast cell activation was demonstrated to occur through P2X_7_ receptors (159). A role for the P2X_7_ receptor in allergic airway inflammation and AHR was demonstrated using P2X_7_ deficient mice and treatment of wild type mice with a specific P2X_7_ antagonists given before each antigen challenge (160). Recently, CD203c or ecto-nucleotide pyrophosphatase-phosphodiesterase 3 (E-NNP3), a widely used activation marker of mouse mast cells and basophils, was demonstrated to negatively regulate IgE-antigen-mediated activation through hydrolyzation of extracellular ATP (161). Further, while E-NNP3^−/−^ mice had exacerbated allergic airway inflammation, mice lacking both E-NNP3 and P2X_7_ had decreased responses to IgE-antigen-mediated activation of FcεRI. Therefore, ATP released by IgE-antigen-mediated activation of FcεRI stimulates mast cell activity through P2X_7_, whereas E-NNP3 decreases the ATP concentration and suppresses mast cell (and basophil) activity *in vivo*.

In human lung mast cells, the expression of the purinergic receptors (P2X_1_, P2X_4_, P2X_7_, P2Y_1_, P2Y_2_) has been confirmed by q-RT PCR and a gene array (162–164). An early study indicated that ATP stimulation of human lung mast cells did not directly induce degranulation but enhanced histamine release after anti-IgE mediated activation (162). The demonstration of P2X_7_ receptor on mast cells in the colon from patients with Crohn's disease (159) suggests that this receptor may regulate mast cell function also in human disease. However, a reduced risk of asthma and asthma severity was detected in children with loss-of-function mutations in the P2X_7_ receptor (165).

Adenosine is directly released from many cell types or generated from extracellular AMP by CD73 (ecto-5′-nucleotidase), and the extracellular levels increase under inflammatory conditions (166). In asthmatics and cigarette smokers, adenosine levels were increased in BAL (167, 168). Further, adenosine provoked bronchoconstriction in asthmatics (169, 170). Adenosine exerts its biological functions binding to four distinct G-protein coupled receptors: A1, A2a, A2b and A3 with different affinity (167). In human lung mast cells, adenosine potentiated IgE-antigen-mediated mast cell activation, thereby increasing the release of mediators such as LTC_4_ and histamine (171). Human and mouse lung mast cells express mRNA for A2a, A2b and A3, and activation via A3 induces histamine release (172–175). In mice, nebulization of an A3 agonist for only 5 min caused mast cell degranulation (175). Moreover, adenosine induced airway contraction, which is lost in mast cell deficient (Kit^*W*/*Wv*^) and in A3^−/−^ mice along with the loss of adenosine-induced mast cell degranulation (176). This suggests that mast cell activation via A3 is the major mechanism behind adenosine-induced bronchoconstriction. In a follow-up study, pre-exposure to aerosolized adenosine was demonstrated to increase methacholine-induced AHR in wild-type, but not in mast cell-deficient (Kit^*W*/*Wv*^ or Kit^*W*−*sh*/*W*−*sh*^) or A3^−/−^ mice (177). Further, the increased AHR after adenosine pre-treatment was regained by reconstitution of Kit^*W*−*sh*/*W*−*sh*^ mice with wild-type BMMCs but not with A3^−/−^ BMMCs. These *in vivo* studies suggest that adenosine can promote bronchoconstriction or enhancing AHR by activating mast cells through the A3 receptor. In the context of human mast cells, adenosine or an A3-specific agonist potentiated FcεRI-induced activation of human lung mast cells (174). Altogether, ATP and adenosine may play an important role in promoting mast cell activation in the allergic lung.

## The Role of Mast Cell Mediators in Allergic Asthma

Many mediators produced upon mast cell activation can be measured in the BAL and other fluids from asthmatic patients as residual levels or after local bronchial allergen challenge (178–181) (Table 2). Here, we summarize what is known about a selected number of mast cell mediators in the context of asthma and *in vivo* models thereof.

**Table 2:** Mast cell mediators detected in elevated levels in patients with asthma and their indicated action in the asthmatic lung.

**Mediator**	**Type of human sample**	**References (e.g.)**	**Actions in the human lung**	**References (e.g.)**
Histamine	BAL (increased after allergen challenge)	(179), (181)	Bronchoconstriction vasodilatation (via H1R) Chemotaxis and modulation of IgE-antigen-mediated activation (via H4R)	(182) (183–185)
Serotonin	Plasma (likely derived from platelets in human asthma)	(186) (187)	Level related with asthma severity AHR	(186) (188, 189)
Tryptase	BAL (increased after allergen challenge) serum (in asthma-related death and severe uncontrolled asthmatics with poor lung function) children with asthma (basal levels)	(181) (190, 191) (192)	Unknown	
Cysteinyl-LTs	BAL (LTC_4_, LTD_4_) Sputum (all) Urine (LTE_4_)	(193) (194) (195)	Vascular leakage Bronchoconstriction airway inflammation (via CysLTR_1_)	(196–199) (200)
LTB_4_	BAL Sputum and exhaled breath condensate	(193) (201)	Chemotaxis of inflammatory cells	(202)
PGD_2_ or its metabolites	BAL (increased after allergen challenge) Plasma/urine (increased after allergen challenge)	(178, 203) (204)	Bronchoconstriction Chemotaxis of eosinophils, basophils and Th2 cells.	(205) (206, 207)

### Histamine

Histamine is a pivotal molecule involved in allergic reactions, and mast cells were recognized early as the main source of histamine (208). In patients with allergic asthma, allergen challenge via bronchoscope stimulates increased histamine levels in BAL (179). Histamine mediates its biological effects by binding to four histamine receptors (H1-4R), which are expressed in many different cell types such as immune cells, nerves and smooth muscle cells (209). In the human airways, the H1R mediates bronchoconstriction and increases vascular permeability (182). Despite this, H1R antagonists have demonstrated variable efficacy in clinical asthma trials (210). In OVA models of allergic airway inflammation, mice lacking H1R had reduced Th2 responses and AHR (211, 212).

Besides mediating the effect on acid production in the stomach, the histamine H2R is associated with the regulation of immune responses (213). A recent study demonstrated that genetic ablation or blocking of H2R in a model of OVA-induced allergic airway inflammation increased the eosinophilia in BAL, type 2 cytokines and mucous production, while an H2R agonist suppressed these responses (214). The exacerbated type 2 inflammation in H2R-deficient lungs was associated with increased numbers of CD1d^+^ dendritic cells and iNKT cells with an increased capacity to secrete cytokines in response to lipid antigens, suggesting that iNKT cell activity is controlled by histamine via H2Rs in the allergic lung.

The H3R is preferentially expressed in the nervous system and regulates its own release from neurons (215). The H4R is the most recently discovered histamine receptor, and like the H1R, this receptor mediates mostly pro-inflammatory effects. The H4R is mainly expressed by immune cells (216). In human eosinophils, histamine potentiated the chemotactic response to eotaxin via the H4R and directly mediated chemotaxis of human basophils *in vitro* (183, 184). In basophils, a specific H4R agonist reduced the mediator release after IgE-antigen-mediated activation (184). The H4R is also expressed in human cord blood-derived and purified lung mast cells (185, 217). In human lung mast cells an H4R-specific agonist induced chemotaxis (185), whereas in cord blood-derived mast cells another selective H4R agonist stimulated degranulation, and generation of lipid mediators and cytokines (217). The H4R is also expressed by mast cells, basophils and eosinophils in mice, and mediates histamine-induced chemotaxis in BMMCs (218). In OVA-induced models of allergic airway inflammation, H4R-deficient and wild-type mice treated with an H4R antagonist during either the sensitization or challenge phase had diminished type 2 inflammation (219, 220). The reduced type 2 lung inflammation in the absence of functional H4R was first attributed to a reduced ability of Th2 cells to produce cytokines (219), and more recently to the fact that dendritic cells cannot be fully activated in the absence of signals through the H4R and therefore the CD4^+^ T cell activation is reduced (220). For detailed information about the history of histamine, its receptors and the use of antagonists, see (221).

### Serotonin

Serotonin (5-hydroxytryptamine, 5-HT) is stored in mast cell granules and released upon mast cell degranulation. However, while mouse mast cells contain large quantities of 5-HT, human mast cells contain none or very small amounts (222, 223). Instead, platelets are the main source of 5-HT in human airways (187). 5-HT has wide biological effects and acts by binding to seven different 5-HT receptor families (5-HT_1−7_R), the serotonin transporter (SERT) and by binding covalently to different effector proteins (224). Although many animal studies have demonstrated that 5-HT induces bronchoconstriction, 5-HT does not consistently induce bronchoconstriction in humans (187). Nevertheless, plasma levels of 5-HT are elevated in asthma patients and are associated with disease severity (186). Further, *ketanserin* decrease adenosine- and methacholine-induced bronchoconstriction in asthma patients (188, 189).

In mice, 5-HT plays a major role in airway contraction mainly via the activation of 5-HT_2_ receptors (225). 5-HT induces a *ketanserin*-sensitive depolarization-response in cholinergic neurons in the trachea, suggesting that 5-HT works by inducing secretion of acetylcholine from nerves that innervate the airways (64). Also, selective 5-HT_2B_R antagonists can block 5-HT-induced bronchoconstriction in mice *ex vivo* (226).

In human airways, 5-HT acts on airway smooth muscle via 5-HT_2A_R, which induces contraction, and via 5-HT_1A_R, which induces relaxation (227). Moreover, 5-HT may also work by inducing or augmenting the release of acetylcholine from cholinergic nerves via 5-HT_3_R and 5-HT_7_R (227). Nevertheless, 5-HT induced eosinophil migration via the 5-HT_2A_R in both mice and humans (228, 229). Mast cells express mRNA for several 5-HT receptors. In a study by Kushnir-Sukhov et al., transcripts for the 5-HT_1A_, 5-HT_1B_, 5-HT_2A_, 5-HT_2B_ and 5-HT_7_ receptors were detected in BMMCs and peripheral blood-derived human mast cells (230). In addition, transcripts for the 5-HT_1E_, 5-HT_2C_, 5-HT_3_ and 5-HT_4_ receptors were also found in peripheral blood-derived human mast cells, while the BMMCs also expressed transcripts for 5-HT_1D_R, 5-HT_6_R and SERT. The same study revealed that 5-HT does not induce degranulation or cytokine release by *in vitro*-derived mast cells from mice or humans. Nevertheless, 5-HT stimulated adhesion to fibronectin and chemotaxis (230). In line with those findings, another study showed that intradermal injection of 5-HT induced 5-HT_1A_R-dependent accumulation of mast cells at the site of injection 48 h post-injection (230).

### Mast Cell Proteases

Mast cell-specific proteases such as tryptase and chymase are stored inside the secretory granules in their active form. In addition, the mast cell granules contain other proteases such as CPA3, also expressed in basophils, as well as cathepsin C and G, found in e.g. neutrophils (231). Upon mast cell degranulation, the proteases are quickly released to the extracellular matrix. Tryptase, which is electrostatically bound to serglycin proteoglycans inside the acidic mast cell granules due to positively charged histidines, will gradually dissociate from the serglycin proteoglycans, possibly forming active monomers before losing its activity through the action of protease inhibitors (232–234). Chymase is also released as a complex with serglycin proteoglycans, but the association is not pH-dependent. Therefore, chymase may stay in complex with serglycin proteoglycan for an extended time and tends to remain close to the mast cell surface after degranulation (235, 236). As described earlier, the expression of tryptase and chymase has been used to classify human mast cells into subtypes. In the mouse, the major form of granule-stored tryptase is mMCP-6. In an acute OVA model of allergic airway inflammation, we found that mMCP-6-deficient mice had reduced methacholine-induced AHR but developed allergic airway inflammation (71). Moreover, the tryptase inhibitors *nafamostat mesylate* and *gabexate mesylate* reduced mast cell activation, AHR and eosinophil influx in an HDM model of allergic airway inflammation (237). A possible mechanism for the pro-inflammatory and broncho-constrictive effects of tryptase is cleavage and activation of protease activated receptor 2(PAR2). When trypsin-like proteases cleave PAR2 in the N-terminal, a tethered ligand sequence is exposed and the receptor auto-activates (238). For example, sensory neurons, which are frequently located in proximity to mast cells, express PAR2 and may thus be activated by mast cell tryptase (239). Moreover, immunostaining shows PAR2 expression in smooth muscle, epithelium, endothelium and glands of human bronchi (240). In the same study, tryptase and an artificial ligand peptide to PAR2 (SLIGKV-NH_2_) were demonstrated to stimulate contraction of isolated human bronchi (240). In mouse models, PAR2 deficiency or blocking PAR2 with a monoclonal antibody reduces allergic lung inflammation and AHR (241–243).

In asthma patients who died due to an asthma attack, tryptase levels in serum were increased compared to those who died from other causes (190). Further, a study of 60 asthmatics classified according to pathological findings and standard clinical parameters to define asthma severity by Bayesian network analysis combined with topological analysis revealed six disease clusters (191). Two of the clusters demonstrated higher serum tryptase levels than the others. One cluster consisted of asthma patients with severe asthma who were generally older, had high BMI, poor lung function and many symptoms, while the other group consisted predominately of patients who were female, obese, non-atopic, had later onset and poor lung function. In children, basal serum tryptase levels were higher in those with mild, moderate or severe persistent asthma than in those with mild intermittent asthma and healthy controls (192). Nevertheless, more studies are needed to clarify the importance of mast cell tryptase and the mechanisms behind its role in allergic and non-allergic asthma.

In mice, the functional homolog of human chymase, mMCP-4, has a protective effect on AHR and lung inflammation. This was shown in two studies, first by analyzing mMCP-4^−/−^ mice in an OVA model of allergic airway inflammation and later in an HDM sensitization model (244, 245). The study by Waern et al. suggested that the protective effect of mMCP-4 is mediated by proteolytic cleavage of IL-33, leading to degradation of this important Type 2 cytokine (245). Moreover, mMCP-4 has the ability to suppress IL-13-induced enhancement of the contraction of the trachea in response to methacholine *in vitro* (246). The protective role of mast cell chymase is in accordance with a clinical study, mentioned earlier, where the number of chymase^+^ mast cells correlated positively with lung function in 20 severe asthmatics (48).

### Lipid Mediators–Leukotrienes

Mast cells are a well-known source of lipid mediators derived from arachidonic acid metabolized by different enzymes in response to various stimuli. Leukotrienes are metabolized from arachidonic acid through the 5-lipoxygenase (5-LO) pathway that generates LTA_4_ as a main precursor of two different types of leukotrienes: the cysteinyl-leukotrienes (cysteinyl-LTs) LTC_4_, LTD_4_ and LTE_4_, and LTB_4_ (247).

The biological activities of the cysteinyl-LTs were discovered more than 30 years ago and included stimulation of smooth muscle contraction and vascular leakage (196–199). The levels of cysteinyl-LTs are increased in BAL (193) and sputum (194) of asthmatics, while LTE_4_ levels are increased in the urine after allergen challenge (195). Inhalation of LTC_4_ or LTD_4_ causes bronchoconstriction, which is 1,000-fold more potent than histamine or methacholine (197–199). The cysteinyl-LTs mediate their biological effects through the receptors CySLTR_1_, CySLTR_2_ and GPR99 (248). Two other receptors, P2Y_12_R and GPR17 are also involved in cysteinyl-LT and LTE_4_-elicited responses, respectively. LTD_4_ has a higher affinity for CySLTR_1_ than LTC_4_, whereas LTC_4_ and LTD_4_ have a similar affinity for CySLTR_2._ However, LTE_4_ has a high affinity for GPR99. CySLTR_1_ is expressed by many different immune cells such as mast cells, neutrophils and monocytes/macrophages, whereas CySLTR_2_ is expressed by eosinophils, macrophages and smooth muscle cells (248). The CysLTR_1_ antagonist, *montelukast*, attenuates CysLT-induced bronchoconstriction and airway inflammation (200), confirming many earlier studies in animal models (249, 250). CysLTR_2_ mediated vascular permeability in IgE-dependent passive cutaneous anaphylaxis and infiltration of macrophages and fibroblasts in a fibrosis model (251). Recently, a role for CysLTR_2_ was demonstrated in IL-33-dependent type 2 immunity (252). Using various knockout strains, CysLTR_2_ was shown to drive IL-33 expression induced by LTC_4_ in two models of allergic airway inflammation. Human cord blood-derived mast cells express both CysLTR_1_ and CysLTR_2_ (253). In response to CysLTR_1_ stimulation, human cord blood-derived mast cells proliferate in a process that is negatively regulated by CysLTR_2_ (254). Moreover, stimulation of the human mast cell line LAD-2 with LTD_4_ or LTE_4_ leads to chemokine and prostaglandin D2 (PGD_2_) release (255).

Inhaled LTE_4_ induces the influx of inflammatory cells in the bronchial mucosa of asthma patients more potently than LTD_4_, while they have similar bronchoconstrictive effects (256, 257). Nevertheless, LTE_4_ has only weak affinity for the earliest discovered cysteinyl-LT receptors, CysLTR_1_ and CysLTR_2_. In mice, P2Y_12_R and platelets were demonstrated to be required for LTE_4_-induced pulmonary inflammation, although LTE_4_ does not bind directly to P2Y_12_R (258). Recently, a placebo-controlled randomized double-blind study of asthmatic patients demonstrated that the P2Y_12_R antagonist *prasugrel* inhibited platelet reactivity but showed only a possible modest effect on mannitol-induced airway reactivity (259). After the discovery of the interaction of LTE_4_-induced responses with P2Y_12_R, another P2Y receptor family member called GPR17 was discovered to function as a negative regulator of CySLTR_1_-mediated responses (260). However, while GPR17 has a role in regulating cysLTR_1_-induced lung inflammation in a HDM model, it is unclear if this effect is directly mediated by Cys-LTs since different labs have conflicting data on whether Cys-LTs can activate GPR17 or not (261). Next, the P2Y receptor family member GPR99 was identified as the third high affinity cysteinyl-LT receptor, which preferentially binds LTE_4_ (262). GPR99 was recently demonstrated to mediate the release of mucin from epithelial cells and swelling of nasal mucosa in mice subjected to a single intranasal dose of *Alternaria* extract or LTE_4_ (263). However, in human asthmatics, LTE_4_-induced bronchoconstriction was completely blocked by the CySLTR_1_ inhibitor *montelukast* (264). The LTE_4_-induced bronchoconstriction was associated with increases in urinary PGD_2_ metabolites and other COX pathway products, which was also abrogated by *montelukast*, suggesting that LTE_4_ activates mast cells (and possibly other cell types) via CySLTR_1_ and that mast cell-derived products activate bronchial smooth muscle cells to constrict. These data suggest that CySLTR_1_ mediates LTE_4_-induced responses in the human lung or that *montelukast* also blocks GPR99.

The non-cysteinyl-LT, LTB_4_, is increased in exhaled breath condensate and sputum of asthmatics (201, 265). In a recent study categorizing asthma patients according to GINA guidelines (step 1–3), patients treated with short-acting beta-agonist (step 1) or inhaled corticosteroids plus long-acting beta-agonist (step 3) had higher LTB_4_ levels in sputum than those treated with inhaled corticosteroids (step 2) (266). This indicates that inhaled corticosteroids reduce LTB_4_ levels in mild-moderate asthma as previously described in (267), but not in more severe asthma. LTB_4_ is produced by various activated leukocytes including mast cells (268), and has a potent chemotactic effect on leukocytes such as neutrophils, T cells and immature BMMCs (202, 269, 270). LTB_4_ mediates its effect through BLT_1_R and BLT_2_R, which are G-protein-coupled receptors. BLT_1_R is a high affinity receptor for LTB_4_ and is responsible for LTB_4_-induced leukocyte migration (202, 248). *In vivo*, intradermal injection of LTB_4_ induced the accumulation of intravenously injected immature BMMCs at the injection site (269).

In experimental allergic airway inflammation, BLT_1_R mediates the infiltration of T cells to the lung (271) as well as AHR, eosinophilic inflammation and goblet cell hyperplasia (272). An indication of a role for mast cells in LTB_4_-mediated allergic airway inflammation was demonstrated in a study using mice lacking LTA_4_ hydrolase (LTA_4_H), which thereby were unable to produce LTB_4_ (273). The LTA_4_H^−/−^ mice had reduced AHR and BAL eosinophilia compared to controls in an active systemic sensitization and challenge model, as well as when mice were passively sensitized and challenged. However, transfer of LTA_4_H^+/+^ BMMCs into LTA_4_H^−/−^ mice normalized airway reactivity to methacholine assessed by electrical field stimulation of tracheal smooth muscle preparations, and partly restored eosinophilia in passively sensitized and challenged mice but not in mice subjected to active systemic sensitization and challenge (273). Thus, LTB_4_ plays a role in allergic airway inflammation, and LTB_4_ from mast cells may mediate some of these effects.

The low affinity LTB_4_ receptor BLT_2_R is expressed in the mouse lung. Intriguingly, BLT_2_R-deficient mice displayed enhanced eosinophilia in an OVA model of allergic airway inflammation (274). This might be explained by the fact that a cyclooxygenase metabolite (*12(S)-hydroxyheptadeca-5Z,8E,10E-trienoic acid*) was shown to have higher affinity than LTB_4_ for BLT_2_R (275). To summarize, leukotrienes and many of their receptors represent important targets in allergic asthma; for a recent detailed review, see (248).

### Lipid Mediators–Prostaglandins

Prostaglandins are metabolites initially derived from the conversion of arachidonic acid by the cyclooxygenases (COX-1 and COX-2) to PGH_2_, which subsequently is a substrate of PGD synthase forming PGD_2_ or PGE synthase forming PGE_2_. There are also other primary prostaglandins (PGF_2α_, PGI_2_, and thromboxane A_2_). However, here we limit the discussion to PGD_2_, which is the major mast cell-derived prostaglandin produced in large amounts through the action of hematopoietic prostaglandin D synthase (HPGDS) in response to IgE-antigen-mediated activation (276). Nevertheless, mouse and human eosinophils also produce PGD_2_ (277). Extracellularly, PGD_2_ is metabolized to different compounds such as 9α,11β-PGF_2_, which can be measured in plasma as a possible indication of mast cell activation (204).

Allergen challenge in allergic asthmatics leads to an increased level of PGD_2_ in BAL, and PGD_2_ metabolites in plasma and urine (178, 203, 204). Inhalation of PGD_2_ leads to bronchoconstriction in normal subjects and to an even stronger bronchoconstrictive response in patients with asthma (205). Despite corticosteroid use, the PGD_2_ synthesis pathway is upregulated in asthmatics with severe and poorly controlled asthma (278). In the same study, the levels of *HPGDS* transcripts in epithelial brushings correlated strongly with the levels of *TPSAB1/TPSAB2* (tryptase) transcripts, suggesting that mast cells are the main source of PGD_2_, at least at this site.

PGD_2_ acts via activation of the D-prostanoid receptors DP1 and DP2. Another name for DP2 is chemoattractant receptor-like protein expressed on Th2 cells, abbreviated CRTH2 (279, 280). The DP1 receptor is expressed on Th2 cells, dendritic cells, basophils, eosinophils, goblet cells and vascular endothelium (276). Recently, mast cell maturation was shown to be mediated by PGD_2_ acting on DP1, a process which was driven by phospholipase A2 group III (PLA2G3) secreted from mast cells, which activated fibroblasts to produce PGD_2_ by the action of lipocalin-type PGD_2_ synthase (281). An amplifying role of PGD_2_ in experimental asthma was discovered by comparing wild-type and mice lacking DP1 in an OVA-induced mouse model of allergic airway inflammation (282). In this study, DP1^−/−^ mice had attenuated AHR and the production of type 2 cytokines was diminished. Further, pre-treatment of sensitized mice with aerosolized PGD_2_ before challenge amplified the type 2 response (283). In contrast, PGD_2_ acts in an anti-inflammatory fashion via DP1 expressed on dendritic cells during the sensitization phase. PGD_2_ or a DP1 agonist, but not a DP2 agonist, instilled intratracheally with FITC-OVA temporarily inhibited the migration of dendritic cells to the lung draining lymph nodes, which limited the expansion of adoptively transferred T cells (284). In a later study by Hammad et al. PGD_2_ or an agonist for DP1 or DP2 was intratracheally delivered 30 min before each OVA challenge in an OVA model of allergic airway inflammation (285). Interestingly, the DP1 agonist (but not PGD_2_ or the DP2 agonist) suppressed inflammation and AHR by a mechanism involving T regulatory cells producing IL-10. As the suppressive effect of the DP1 agonist could be mimicked by adoptively transferred dendritic cells pre-treated with the DP1 agonist. Hence, the DP1 agonist likely acted on lung dendritic cells which acquired a regulatory function by stimulating the expansion of T regulatory cells producing IL-10 (285). DP2/CRTH2 is expressed by various immune cells such as eosinophils, basophils, Th2 cells and ILC2s (286, 287). Moreover, *Ptgdr2* transcripts have been found in BMMCs (288), and CRTH2 immunopositive human mast cells were found in nasal mucosa (289) and nasal polyps (290), although the expression seemed to be intracellular. Early studies demonstrated that CRTH2 mediates chemotaxis of human eosinophils, basophils and Th2 cells (206, 207). Further, patients with severe asthma have an increased level of *CRTH2* transcripts in BAL cells compared to patients with mild to moderate asthma and heathy controls (278). In the same study, immunohistochemical analyses of CRTH2^+^ cells in BAL revealed a higher percentage of CRTH2^+^ cells in asthma patients compared to healthy controls, with the highest percentages found among those with severe asthma and as well as those with mild asthma but who did not have corticosteroid treatment (278).

In mice, sensitization with OVA followed by nebulization of a CRTH2 agonist before each challenge enhanced AHR and the eosinophilia in the BAL and lung tissue (291). In contrast, mice lacking *Ptgdr2* have an enhanced BAL eosinophilia in OVA-induced allergic airway inflammation (292). This was likely due to the fact that T cells lacking CRTH2 produced higher levels of cytokines (292). However, a CRTH2 antagonist given in connection with OVA challenge in a model of allergic airway inflammation reduced eosinophilia and mucus hyperplasia (293). Similarly, mice sensitized and challenged with cockroach allergen given a CRTH2 antagonist before the final challenge had significantly reduced AHR, levels of type 2 cytokines in the lung and allergen-specific IgE and IgG2a in serum (294). For these reasons, CRTH2 antagonists have been tested as a treatment for asthma. Several different CRTH2 antagonists have been taken into clinical trials, but some of them have been discontinued for various reasons like poor effect or pharmacokinetics (295). However, other CRTH2 antagonists have been demonstrated to improve lung function, symptom scores and reduced sputum eosinophilia (296–298). Still more studies are needed to define what asthma phenotypes will benefit (most) from treatment with a CRTH2 antagonist.

### Lipid Mediators–Sphingosine-1-Phosphate

A more unknown lipid mediator that is generated and released from mast cells after IgE-antigen-mediated activation is sphingosine-1-phosphate (S1P) (299). S1P is produced as a result of sphingosine (Sph) phosphorylation by two Sph kinases (SphK1 and SphK2). In mast cells, IgE-antigen-mediated crosslinking of FcεRI induces the activation and translocation of SphK1 to the plasma membrane and leads to increased levels of S1P, which then becomes secreted extracellularly (299). S1P is produced by most cell types but is usually degraded or dephosphorylated intracellularly, and the level in the body is tightly regulated (300). S1P binds to five different receptors (S1PR1-S1PR5) expressed by both innate and adaptive immune cells. The main outcome of S1P binding to these receptors seems to be directed migration. In patients with asthma, the S1P concentration is increased in BAL after allergen challenge (301). In the same study, stimulation of human airway smooth muscle cells by the S1P-activated signaling pathways was involved in contraction, proliferation and stimulated IL-6 release. In addition, S1P induced contraction of human airway smooth muscle cells embedded in collagen matrices (302).

In mice, an inhibitor of Sph kinases given before each OVA challenge in a model of allergic airway inflammation reduced inflammatory BAL cells and type 2 cytokines, AHR and mucus production (303). In isolated bronchi from mice, S1P enhanced acetylcholine-induced contraction (304). Further, S1P induced contraction of OVA-sensitized lungs but not in naïve lungs as demonstrated both in isolated bronchi and *in vivo* measured by whole-body plethysmography (304). In a follow-up study, Roviezzo et al. demonstrated that subcutaneous administration of S1P day 0 and day 7 dose-dependently increased bronchial responsiveness *in vivo* and *ex vivo* (305). The effect was time-dependent, with the greatest effect found 21 days after S1P administration. At this time point the number of mast cells quantified in BAL was twice that found in the vehicle controls. Using the same protocol of S1P-induced asthma-like inflammation, the same authors recently demonstrated that while LPS potentiated S1P-induced AHR, TLR4-defective mice (C3H/HeJ) or BALB/c mice pre-treated with an TLR4 blocking antibody were protected from S1P-induced AHR (306). Moreover, S1P induced higher expression of TLR4 in the proximity of the bronchi, and immunoprecipitation revealed an increased association between S1PR1 and TLR4. Together, this study suggests a functional interaction between S1PR1 and TLR4 that amplifies allergic airway inflammation and airway reactivity.

Mast cells were implicated in S1P-mediated responses *in vivo* in a model of IgE-mediated systemic anaphylaxis. In this study, pre-treatment with a S1P neutralizing antibody delayed and reduced peri-vascular lung inflammation along with BAL tryptase and histamine serum levels in mice injected intraperitoneally with dinitrophenyl (DNP)-specific IgE 12 h before injection of DNP coupled to human serum albumin (307). In the same model, mast cell deficient mice (Kit^*W*−*sh*/*W*−^^sh^) or mice pre-treated with an S1PR2 antagonist or an anti-S1P antibody had reduced early peri-vascular lung inflammation. This suggests that mast cells providing and/or responding to S1P via S1PR2 are responsible for early peri-vascular lung inflammation observed in the model of IgE-mediated systemic anaphylaxis. In humans, an inhibitor of all S1PRs except S1PR2 (FTY720, *fingolimod*) is approved as a drug to treat multiple sclerosis (308). This drug was tested on moderate asthmatics in a double-blind, placebo-controlled 10-day study (309). Patients that received high doses of *fingolimod* showed only mild reductions in some lung function parameters and needed more short acting beta agonists, perhaps suggesting that although S1PR2 may be a target for asthma, the other S1P-receptors should not be targeted.

### Cytokines

Cytokines play a crucial role in coordinating and maintaining allergic inflammation in the asthmatic lung. For a recent update on the general importance of cytokines in asthma, please see (310). Mast cells have the ability to secrete a broad spectrum of cytokines e.g. IL-1β, IL-2,-3,-4,-5,-6,-9,-10,-11,-12,-13, TNF-α, IFN-γ, GM-CSF, SCF, and TGF-β, as recently reviewed (311). In asthma patients, mast cells have been demonstrated to produce and release IL-4 (312). Interesting to note is that mouse MCp also have the ability to produce IL-4 (313). Possibly, this Th2-skewing cytokine derived from mast cells may promote or support early Th2-differentiation, although the contribution of mast cell-derived IL-4 in asthmatic lungs is unknown. Tryptase^+^ mast cells producing IL-4, as well as IL-5, IL-6 and TNF, were found in the bronchial mucosa from both healthy and asthmatic subjects (314). Moreover, isolated human mast cells produce IL-13 upon IgE-crosslinking (315), and IL-13 and IL-4 expressing mast cells are found within the airway smooth muscle of asthma patients (316). In mice, a role for IL-13, FcεRI and mast cells in the development of AHR was demonstrated in a model of OVA-induced allergic airway inflammation (77). Human lung mast cells also produce TNF-α (317). As already described, mast cell-derived TNF-α has been suggested to contribute to the development of allergic airway inflammation, Th2/Th17 cytokine production and AHR in mice (70, 73).

IL-9 is a pleiotropic cytokine, produced by many types of immunes cells including mast cells (318). Genetic studies have revealed that the IL-9 receptor is associated with asthma susceptibility (319, 320), and elevated IL-9 mRNA and IL-9 immunoreactive cells are found in lungs from asthmatics compared to patients with chronic bronchitis, sarcoidosis and healthy controls (321). In mouse models, lung selective IL-9 overexpression leads to mast cell accumulation, along with massive airway inflammation and AHR in the absence of an antigen (322). Nevertheless, as reviewed in (323), there is some conflicting evidence for the significance of IL-9 in mouse models of asthma, indicating that an alternative pathway exists. In the context of allergic asthma, mast cells responding to IL-9 signals are likely more important than mast cells as a source of this cytokine, as discussed earlier and demonstrated in different experimental set-ups of allergic lung inflammation in mice. In principle, IL-9 seems to be required for antigen-induced accumulation of lung mast cells in mice. Interestingly, a humanized anti-IL-9 monoclonal antibody (MEDI-528) has been tested on 36 mild asthmatics (injected subcutaneously) (324). The results demonstrated a trend toward a protective effect of MEDI-528 on lung function (324). However, in a randomized, controlled trial of 329 patients in which MEDI-528 (or placebo) was added to their usual asthma treatment over 24 weeks, no improvements in quality of life, asthma exacerbation rates or FEV_1_ values (pre-bronchodilator) were found (325). Thus, the future of targeting IL-9 in asthma is unclear although it is still possible that this pathway may be a valid target for a subgroup of asthma patients. To summarize, mast cells can produce many different cytokines that have been demonstrated to play significant roles in allergic asthma. However, whether or not the mast cell-produced cytokines play a major critical role in the human disease is currently unclear.

## Conclusion

To conclude, asthma is a heterogenous disease highlighted by the differences in, e.g. time of on-set, severity, inflammatory pattern and responsiveness to corticosteroid-treatment. Similarly, we imagine that asthma patients differ in whether or not mast cells play a major or minor role in the pathogenesis. Given the plentitude of mediators that mast cells secrete, the limited beneficial effect of histamine receptor antagonists it is not surprising. It will likely be impossible to efficiently treat allergic asthmatics by targeting another single mast cell mediator. Still, the success of *Omalizumab* as a treatment for a subgroup of allergic asthmatics with severe or persistent symptoms indicates an IgE-mediated mast cell-involvement in their disease (104–106). Thus, finding out what other triggers of mast cell activation that may cause symptoms in different sub-groups of allergic asthmatics would be a better strategy for the development of novel drugs targeting mast cells.

We find that recent advances suggest that resident CTMCs are mainly self-sustained and of fetal origin. In the lung of patients with allergic asthma, mast cells accumulate in the smooth muscles, bronchial epithelium and alveolar parenchyma. The unique location of these lung mast cells can be replicated using mouse models of allergic asthma, and in these models the accumulation of mast cells is preceded by the recruitment of MCps to the lung. Although studies using mast cell-deficient mice subjected to allergic airway inflammation protocols have defined many new possible mechanisms that may occur in the human disease, these mechanisms need to be re-evaluated using the new *Kit*-independent mast cell-deficient strains in an attempt to clarify the role of mast cells in allergic airway inflammation.

## Author Contributions

EM-E and JH conceptualized, wrote, and edited the manuscript. EM-E designed the study. JH edited the figures.

### Conflict of Interest Statement

The authors declare that the research was conducted in the absence of any commercial or financial relationships that could be construed as a potential conflict of interest.
